# Rapid Prototyping for Nanoparticle-Based Photonic Crystal Fiber Sensors

**DOI:** 10.3390/s24123707

**Published:** 2024-06-07

**Authors:** Michael Sherburne, Cameron Harjes, Benjamin Klitsner, Jonathan Gigax, Sergei Ivanov, Edl Schamiloglu, Jane Lehr

**Affiliations:** 1 Johns Hopkins University Applied Physics Laboratory, Laurel, MD 20723, USA; 2 Air Force Research Laboratory, Albuquerque, NM 87117, USA; cameron.harjes@us.af.mil; 3 Center for Integrated Nanotechnologies, Albuquerque, NM 87123, USA; bhklits@sandia.gov (B.K.); jgigax@lanl.gov (J.G.); ivanov@lanl.gov (S.I.); 4 Department of Electrical and Computer Engineering, University of New Mexico, Albuquerque, NM 87131, USA; edls@unm.edu (E.S.); jmlehr@unm.edu (J.L.)

**Keywords:** additive manufacturing, cleaving, photonic crystal fiber, THz laser

## Abstract

The advent of nanotechnology has motivated a revolution in the development of miniaturized sensors. Such sensors can be used for radiation detection, temperature sensing, radio-frequency sensing, strain sensing, and more. At the nanoscale, integrating the materials of interest into sensing platforms can be a common issue. One promising platform is photonic crystal fibers, which can draw in optically sensitive nanoparticles or have its optical properties changed by specialized nanomaterials. However, testing these sensors at scale is limited by the the need for specialized equipment to integrate these photonic crystal fibers into optical fiber systems. Having a method to enable rapid prototyping of new nanoparticle-based sensors in photonic crystal fibers would open up the field to a wider range of laboratories that could not have initially studied these materials in such a way before. This manuscript discusses the improved processes for cleaving, drawing, and rapidly integrating nanoparticle-based photonic crystal fibers into optical system setups. The method proposed in this manuscript achieved the following innovations: cleaving at a quality needed for nanoparticle integration could be done more reliably (≈100% acceptable cleaving yield versus ≈50% conventionally), nanoparticles could be drawn at scale through photonic crystal fibers in a safe manner (a method to draw multiple photonic crystal fibers at scale versus one fiber at a time), and the new photonic crystal fiber mount was able to be finely adjusted when increasing the optical coupling before inserting it into an optical system (before, expensive fusion splicing was the only other method).

## 1. Introduction

Nanoparticles may play a critical role in shrinking the size of a variety of sensors. Optical nanoparticles, such as quantum dots, have utility in temperature sensing [1], ionizing radiation detection [2], and the measurement of high-power electromagnetic fields when combined with magnetic nanocrystals [1,3,4,5,6]. Bifunctional nanoparticles, using multiple families of nanocrystals, were demonstrated and their combination with optically active materials and those affected by other fields is expected to expand in the future [7,8,9,10]. A promising approach is to use holey fibers—a type of photonic crystal fibers (PCFs)—to draw in colloidal nanoparticle solutions by capillary action and allow these particles to have their optical emission guided in a fiber device. Even with accessibility to a fusion splicer to produce multimode (MM) fibers from PCFs, this process is imprecise. It is complicated to properly fuse the tips of the PCF to an MM fiber without overmelting the air capillaries. Their air gaps provide an excellent medium to draw in colloidal nanoparticle solutions and allow these particles to have their optical emission guided in a fiber device. For most laboratories, accessibility to a fusion splicer to fuse PCFs to multimode (MM) fibers is not an option. Even with a fusion splicer, fusion splicing can be imprecise, making it complicated to properly fuse the tip of the PCF to the tip of an MM fiber without overmelting the air capillaries of the PCF. Moreover, PCFs are difficult to conventionally cleave with a diamond blade. Here, we present a method to reliably cleave PCFs with a consistently clear surface. The process is scalable and drastically reduces the time spent on quality assurance tasks on the fiber faces and cleaves. Finally, drawing colloidal nanoparticles through PCFs is also non-trivial and procedures for performing this at scale for the mass development of such sensors has not been demonstrated. Typically, a vacuum can be applied to a single PCF to draw a colloidal nanoparticle solution through, but this can come with a safety concern if a seal has a leak, drawing in oxygen into a Schlenk line. The oxygen will be condensed and turn into liquid oxygen, which is not easy to deal with.

The work discussed in this article involved the development of a new method for integrating nanoparticle-based PCFs into an MM fiber system. Magnetite nanocrystals were used to demonstrate the drawing process of the PCFs.

This manuscript is organized as follows: The initial theory explaining how PCF sensors work is explained. In addition, mechanisms are summarized regarding why magnetite PCF sensors change the optical transmission of the signal when under a magnetic field and when experiencing changes in temperature. Next, we provide the methodology going over the novel process in reliably cleaving PCFs, the 3D-printed part used for vacuum drawing PCFs, and the 3D-printed part used for securing PCF sensors for running sensor experiments. An example experimental setup utilizing the new PCF processing methodologies proposed in this paper is provided in Appendix D. This appendix showcases the strengths of the PCF processing methodologies when running a PCF-drawn nanomaterial experiment. Finally, concluding remarks on this new nanoparticle sensor fiber integration methodology and recommendations for improvements are given.

## 2. Theory

Before discussing how to integrate nanoparticles into a PCF optical system, a summary of how various types of nanoparticles can be used for sensing in such a system is explained. Previous literature shows that PCFs with magnetic ferrofluid (ferrous nanoparticles) change their optical transmission under changes in both temperature and direct current (DC) magnetic fields [1,3,4,5,6,11,12,13] by altering the scattering properties of the ferrofluid. The changing scattering properties of the ferrofluid change the index of refraction of the PCF material [1]. For endlessly single-mode PCFs, they guide light through a total internal reflection guide. As a magnetic field is applied, the ferrous nanoparticles will begin to physically shift, which changes the refractive index properties of the fluid [3]. Chen et al. demonstrated that a PCF’s optical transmission is sensitive to a ferrofluid covering its outside surface [11]. Since the magnetite nanofluids are drawn internally, one would not need to worry about this surface effect.

Magnetite nanocrystals could also interact with a PCF in another way. When one bakes out the ferrofluid within the PCF, it will then deposit the magnetite nanoparticles as a thin film around the surface of the capillaries within the PCF. When applying magnetic fields at certain frequencies, the magnetite nanoparticles will heat up, which, in turn, will begin to change the temperature of both the magnetite’s surfactant ligands (organic molecules) and the PCF cladding and, therefore, change the optical properties of the PCF (birefringence) [14]. Evanescent waves can be affected by magnetite ferrofluid solutions, which, in turn, would also affect the optical transmission response. Previous literature for a different application have used nanomaterials to affect optical light within the capillaries using the evanescent waves being focused around the PCF’s core [15]. Thus, the evanescent wave theory for the dried magnetite affecting the optical transmission under magnetic field irradiation is plausible. However, another plausible hypothesis is that the photonic crystal’s index guiding abilities in a PCF can be affected by a change in the localized temperature of the capillaries. These capillaries help to act as an optical barrier to guide the light through the core of the PCF. Localized changes in temperature from materials within the capillaries and the capillaries themselves can change the guiding properties of the light, which, in turn, can affect the optical transmission intensity.

**Figure 1:**
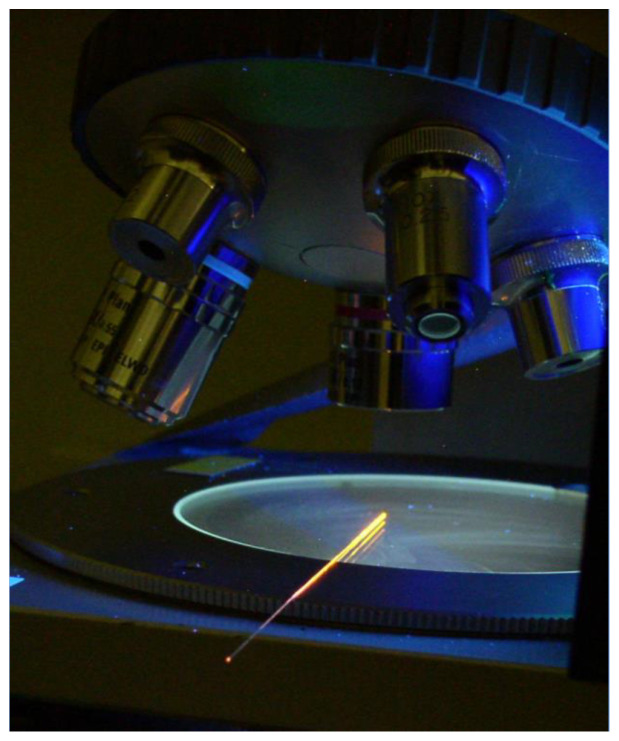
UV excitation of CQDs in fiber from the side; notice the light emission output toward the bottom-left of the fiber [16].

Another use of nanoparticles within a PCF was discussed in the 2014 report by Burke et al. [16] who investigated colloidal quantum dots (CQDs) for X-ray imaging. They looked at a few methods to integrate CQDs for the application, and one of their most unique ideas was drawing CQDs into a PCF [16]. The CQDs were small enough to be drawn through the PCF via capillary action. The NASA team also used a small pressure gradient to help ease the drawing process [16]. An image showing the light emission of the CQDs within the PCF from the NASA team can be seen in Figure 1.

From Figure 1, it can be seen that UV stimulation of the CQDs can occur isotropically due to some of the light leaking out of the sides of the fiber by not meeting the angle needed for total internal reflection (TIR). However, the photons that meet the TIR angle become anisotropic when coming out of the fiber through waveguiding. As mentioned in the NASA presentation, the CQD-infused fibers can be spliced into a main fiber to guide the light to a sensor [16].

Another useful application of nanomaterials drawn through a PCF could be for the advancement of pulsed fiber lasers. Researchers previously looked into PbS/CdS CQDs within a polyvinyl alcohol (PVA) polymer as a saturable absorbers (SAs) [17]. However, there are inherent issues in transitioning it. Other researchers looked into doping glass with near-infrared (NIR) absorbing CQDs and other polymers [18,19,20]; yet, they ran into an issue with being integrated into a fiber laser system with low coupling loss. Instead of having NIR-absorbing CQDs within other materials that have inherent challenges in being integrated into a laser fiber system, we can instead draw up these CQDs within a photonic crystal fiber (PCF) and directly integrate them into a fiber laser system. However, the scalability of cleaving fibers, drawing them with nanocrystals, and combining them into a laser system while testing multiple nanomaterial parameters is an issue. This study’s methodology was intended to help make this development easier.

## 3. Materials and Methods

This section first discusses the magnetite nanomaterials used as an example material for drawing nanoparticles through a PCF, the new process for cleaving PCFs, the 3D-printed pressure adapter, the 3D-printed connector supporting structure, and the experimental design to test the magnetic and thermal responses of the magneto-optic sensors.

### Magnetite Nanoparticles Used and Their Characterization

**Figure 2:**
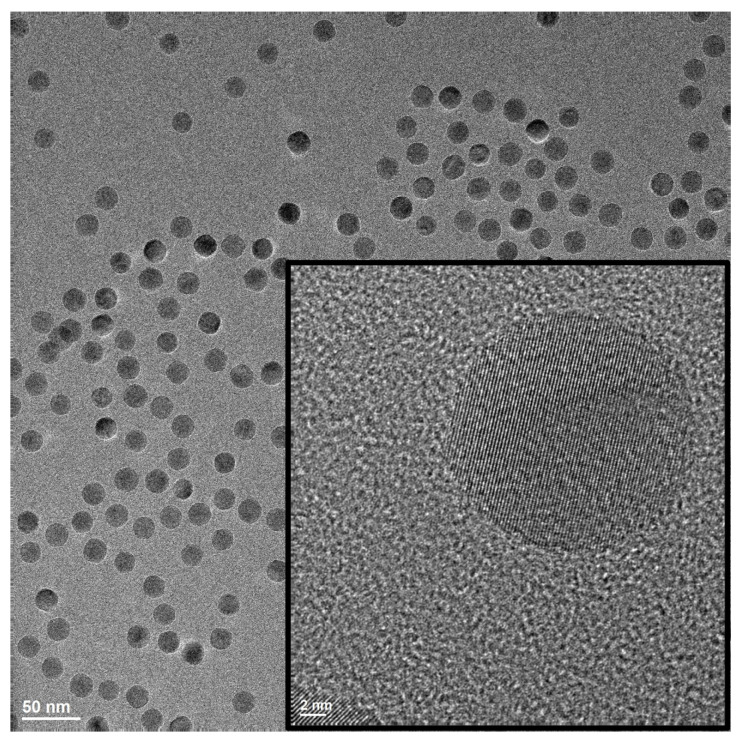
TEM image for 20.2 nm average diameter magnetite nanoparticles.

Magnetite nanoparticles were made using the process presented in Vreeland et al.’s article [21]. The work described in this article used magnetite nanoparticles synthesized with an average diameter of ≈20 nm. The size distributions were determined by using small-angle X-ray scattering (SAXS). Before taking the SAXS measurements, each sample was filtered through a 0.22 μm filter. From the best-fit curves for the SAXS data, the average diameter was 20.2 nm and the dispersion was 4%. The SAXS curve can be seen in S1. A Technai transmission electron microscope (TEM) image taken of the magnetite nanocrystal can be seen in Figure 2.

**Figure 3:**
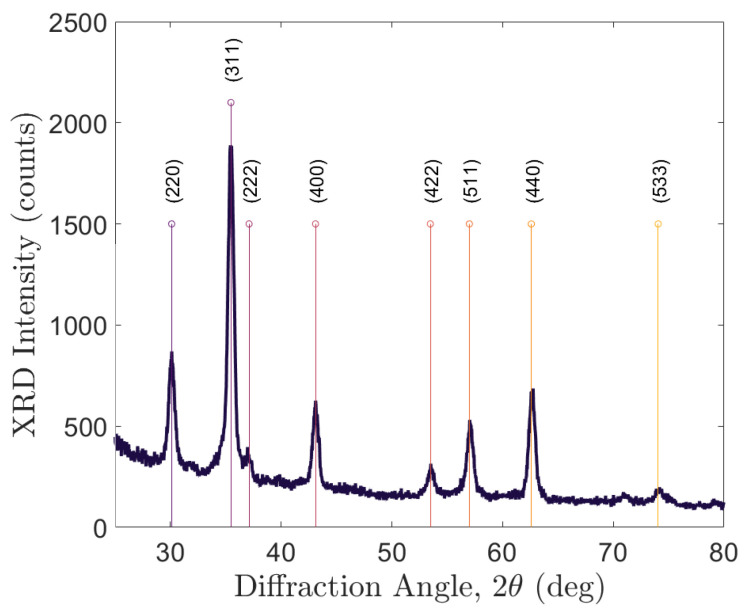
XRD measurements of the magnetite nanocrystal samples showing peaks at 30.1°, 35.5°, 37.1°, 43.1°, 53.5°, 57.0°, 62.6°, and 74.1°. These peaks correspond to ${\mathrm{Fe}}_{3}O_{4}$ material.

To confirm that the materials from the mentioned synthesis process were ${\mathrm{Fe}}_{3}O_{4}$, a SmartLab II Rigaku with Cu $K_{\alpha }$ radiation was used to take X-ray diffraction (XRD) measurements. This diffraction pattern can be seen in Figure 3.

Along with confirmation from the PDF4+2022 materials database, the peaks in the XRD were the same for magnetite nanoparticles, as seen in other publications [4,22,23]. The concentration of magnetite in the sample was determined by using the following process: (1) the Fe_3_O_4_ solution was vibrated in hexane for 30 s; (2) the material was sonicated for 10 min; (3) the empty crucible was weighed; (4) 0.2 mL of material was drawn out and dispensed into an empty crucible; (5) the filled crucible was weighed; (6) a colloidal sample was placed in a crucible under nitrogen air to dry out; (7) the dried sample was weighed in the crucible; (8) the crucible was placed into a thermogravimetric analyzer (TGA) and the organic matter was burned off in a 100% nitrogen environment, stopping at 400 °C (this mitigated soot build-up); and finally, (9) the final weight measurement of the sample in the crucible in the TGA was obtained. One could then use the final weight measurement and divide by 0.2 mL to obtain the approximate concentration of magnetite. Any residual soot was assumed to be negligible. For the magnetite stock used to load into the PCFs for this article, the magnetite nanoparticle solution had a concentration of 9.25 mg/mL.

## 4. Novel Photonic Crystal Cleaving Process

**Figure 4:**
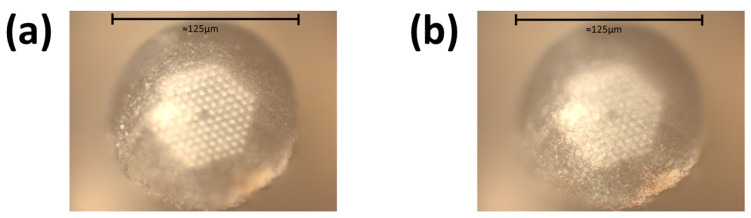
(**a**) Focal plane set to the surface of a PCF. (**b**) Focal plane set to the edge of the redepositioned glass.

Conventional cleaving of a PCF using a scoring blade is not the most reliable technique and does not result in the best surface finish (some defects are left behind) [24]. This paper shows a new process that guarantees a well-cut fiber tip every time (smooth surface finish and all holes open) and relatively easy to scale. It uses a femtosecond pulsed laser (model: Coherent Monaco 1035, Coherent, Inc., Santa Clara, CA, USA) to cut through the fiber with a beam diameter of ≈20 μm after being focused through a 5× microscope objective. This diameter was confirmed with a burn test on a piece of copper. This was followed by a plasma-focused ion beam (PFIB) (ThermoFisher Helios G4, Thermo Fisher Scientific, Waltham, MA, USA) Xe ion source to clean off the surface of the fiber tip of redeposited glass. We termed this novel process as the laser-cleaving plasma-cleaning (LCPC) method. Femtosecond pulsed lasers have inherent issues when it comes to precision cutting at the micrometer scale. As the laser interacts with the surface it is ablating, it produces a plasma plume that contains heated material. This heated material can redeposit itself along the surface the laser is cutting through. This is problematic for PCFs, which have fine microchannels that need to be clear in order to allow for the drawing of nanoparticles. Hence, this redeposited glass needs to be cleared away. An example of this redeposition of material can be seen under a microscope in Figure 4.

**Figure 5:**
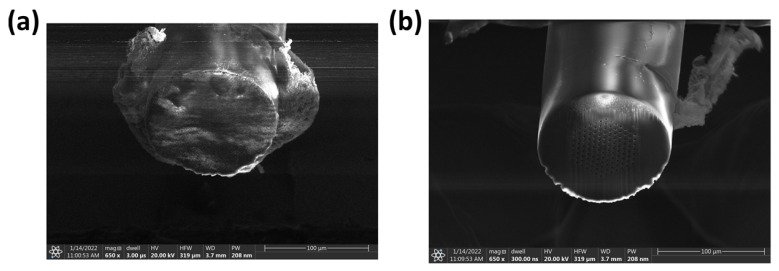
SEM images taken after the femtosecond laser cutting and the PFIB plasma-cleaning processes. (**a**) Shows the PCF after being cut by a femtosecond pulsed laser and (**b**) shows the same PCF having the redeposited glass being cleaned up by a PFIB.

A legitimate concern is the redeposition of material within the microchannels of the PCF, hence this methodology requires using the PFIB to etch away enough of the PCF to eliminate any areas with redeposited material that could have gone into the microchannels. The redeposition of material, as seen in Figure 4, is a legitimate concern for the proper waveguiding of a PCF when connected to another optical fiber. The additional step of a PFIB is then used to etch away this redeposited glass layer. An example of this whole process could be seen using a scanning electron microscope (SEM), as shown in Figure 5.

The PCF used in this work was a NKT Photonics LMA-8 in endlessly single mode. This PCF was entirely made of pure silica for its core and cladding material and its single coating layer was made of acrylate. An SEM image of the fiber end was taken and ImageJ post-processing was used to measure the approximate dimensions of the PCF. The inner hole diameters were ≈2.2 μm. The hole-center-to-hole-center distance was ≈5.4 μm and there were 138 holes within the PCF’s core. The sub-micrometer-sized holes were large enough for the magnetite nanocrystals to be drawn through.

There is an optimal setting to cleaving the PCF using a femtosecond pulsed laser as too aggressive of a cut can lead to major deformations of the surface. The tip of the PCF needs to be as intact as possible to ensure proper waveguiding and the structural integrity of the microchannels. First, a long length of fiber was loaded into a FemtoScribe system. The laser was set to cut the fiber over 10–20 passes. The laser was set at an output wavelength of 1035 nm and a pulse duration of 350 fs. The additional laser parameter settings used to home in on the optimal cutting can be seen in Table 1.

**Figure 6:**
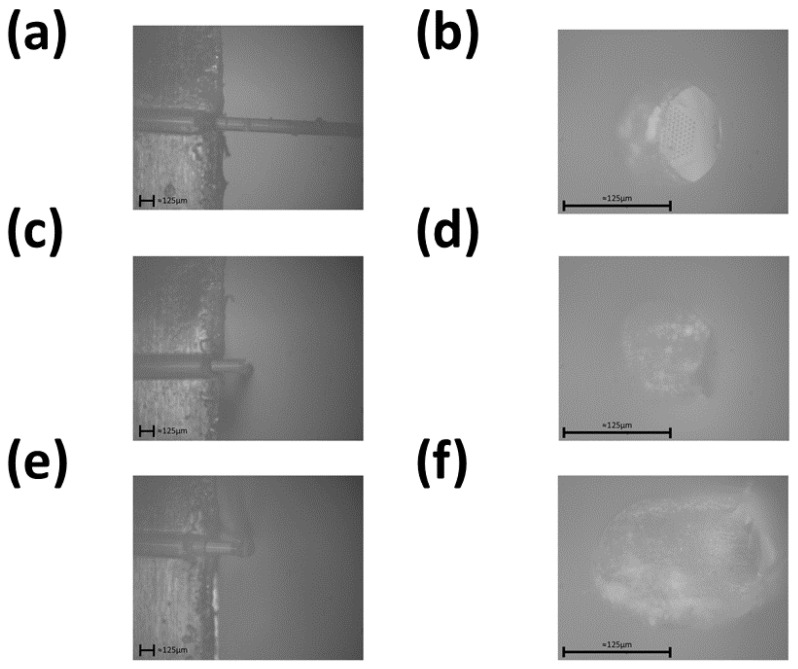
(**a**) Side view microscope image of test one. (**b**) Top view microscope image of test one. (**c**) Side view microscope image of test two. (**d**) Top view microscope image of test two. (**e**) Side view microscope image of test three. (**f**) Top view microscope image of test three.

The observed SEM image results of tests one through three from Table 1 can be seen in Figure 6.

**Figure 7:**
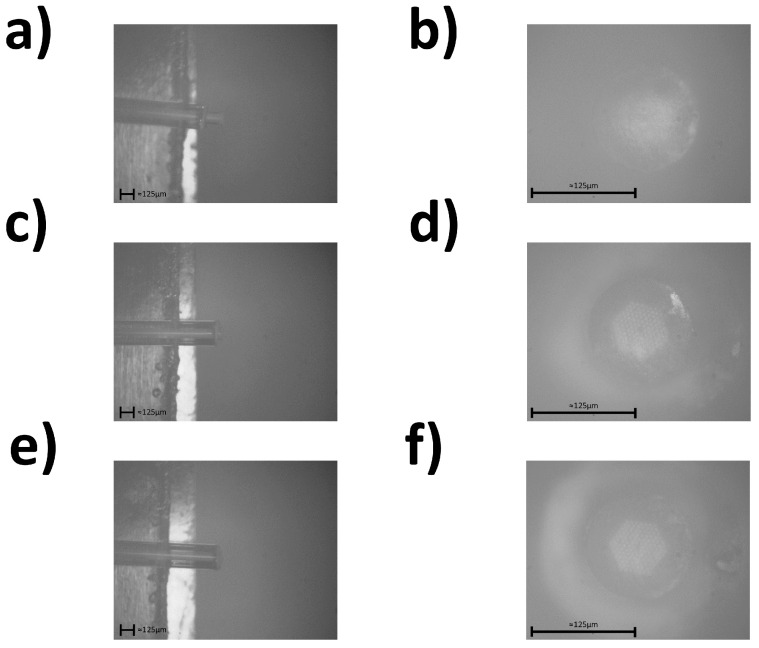
(**a**) Side view microscope image of test four. (**b**) Top view microscope image of test four. (**c**) Side view microscope image of test five. (**d**) Top view microscope image of test five. (**e**) Side view microscope image of test six. (**f**) Top view microscope image of test six.

As can be seen from Figure 6, the increase in power and repetition rate created a larger amount of redeposition of silica and deformation of the PCF tip. Thus, the following three tests focused on a lower power and faster stage speeds. The observed SEM image results of tests four through six from Table 1 can be seen in Figure 7.

Test six from Figure 7 was shown to be the ideal set of laser-cleaving parameters. Test four’s slow stage speed created a thick redeposited silica layer. Test five’s RF power was too strong, resulting in a significant surface defect of the PCF’s tip. While the PCF tip may look great under an SEM, there still existed a layer of redeposited silica over the microchannels. Next, a PFIB (model: ThermoFisher Helios G4) with a xenon ion source was used to mill down 0.2 mm–0.3 mm of the surface of the PCF to remove this redeposited silica layer and open up the microchannel holes. The PCFs were secured inside the PFIB using copper tape. The PFIB was set to the following parameters: electron voltage of 30 kV and a source current of 2.5 μA. The final result can be seen in Figure 5b.

**Figure 8:**
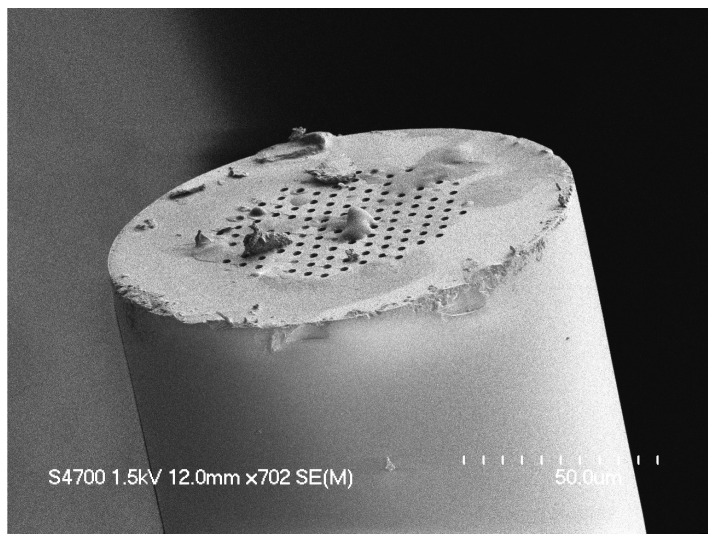
SEM image of a PCF face after conventional cleaving with small defects affected some of the holes of the fiber. Small defects as pictured here can be difficult to see under a standard light microscope during faster quality assurance [24].

The yield success rates following this LCPC process became 100% versus using conventional cleaving tools that were more likely to leave defects that clogged a PCF hole. One such cleaving defect that is difficult to determine under a microscope from conventional methods can be seen in Figure 8.

**Figure 9:**
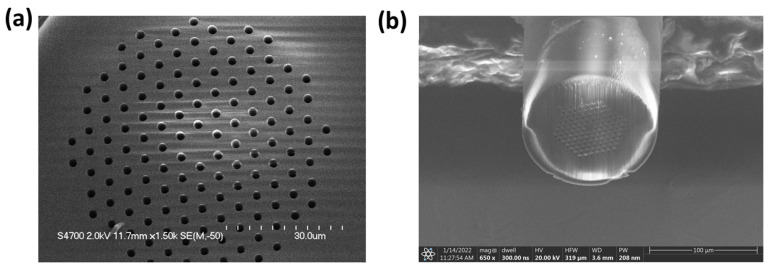
(**a**) PCF face cleaved with a diamond blade cleaver [24]. (**b**) PCF face cleaved using the LCPC method.

In addition, this LCPC method left no shockwave ripple surface artifact across the surface of the PCF, whereas cleaving always left shockwave ripples across its face due to how traditional diamond fiber cleaves make use of a shockwave to make a “smooth” cut. This comparison can be seen in Figure 9.

Overall, the LCPC method can be the preferred way to make batches of cut PCF without having to worry about any surface defects potentially clogging the holes of the PCF. In addition, an assured flat surface can be better for coupling the PCF to another fiber. This leads to significant cost savings in terms of both funding and time of personnel when getting PCFs ready for the loading of nanoparticles, as a quality assurance check is not needed for each cleaved PCF. The significant factor is that the LCPC method can be scaled to process numerous PCFs at once. It should also be noted that any pulse duration in the 10s of picoseconds range (above 20 ps) can be used to do the laser ablation of silica (the fiber material in this paper). The pulse duration in this study was set by the pulse duration of the laser used in this study. Anything longer in pulse duration would cause cracking of the ceramic fiber induced by high thermal transients in the system [25,26].

## 5. Three-Dimensional-Printed Fiber Pressure Adapter

**Figure 10:**
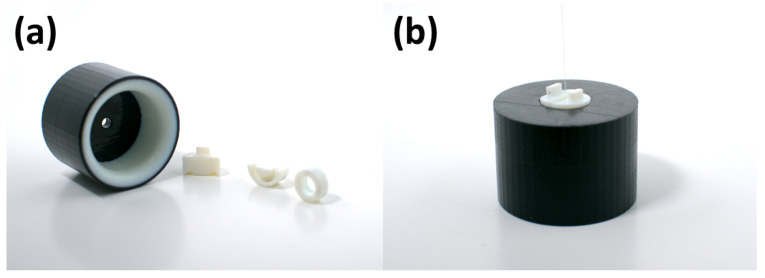
(**a**) Individual components of the 3D-printed pressure adapter. (**b**) Assembled components of the 3D-printed pressure adapter with a PCF attached. This could then be inserted onto the neck of a flask.

A specialized 3D-printed part was developed to easily apply positive pressure to a PCF when drawing up colloidal nanoparticles. It consisted of an interface using an RGD852 Vero Magenta V by Stratasys (Eden Prairie, MI, USA) and two rubber clamps with a rubber o-ring using an Agilus30 White FLX945 by Stratasys. These parts were all printed using a Stratasys J850 PolyJet 3D printer. This printer was used for the rest of 3D-printed parts in this study. The 3D-printed fiber pressure adapter can be seen in Figure 10. Orthographic drawings can be seen in Appendix B.

**Figure 11:**
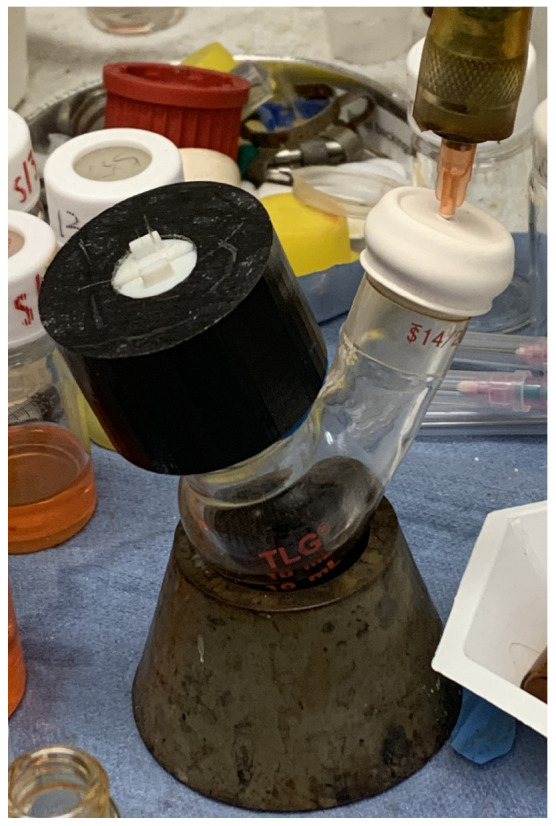
Two-neck flask setup consisting of a nitrogen line connected to a syringe needle inserted into a septum on the angled neck. The nanomaterial was loaded into the flask. The septum was secured with super glue. The 3D-printed PCF loaded was inserted onto the vertical neck with one end of the PCF sitting within the nanomaterial solution. Fun-tak was used to create a better air seal around the bottom of the 3D-printed PCF loader.

In order to use this pressure adapter, we first added in anywhere from one to three PCFs on one of the rubber clamps that was over a rubber o-ring. These PCFs were pushed through the hole in the pressure adapter. Then, a second rubber clamp was carefully slid onto the pressure adapter in order to make a seal. The pressure adapter was then slid into the top neck of the flask. The second neck of the flask had 5 SLPM $N_{2}$ of positive pressure applied using a syringe piercing through a septum on the neck of the flask. Super glue was used to create an airtight seal around the syringe. The tip of the PCF fiber was submerged into the colloidal magnetite nanoparticle solution on top of a non-magnetic surface. If this were non-magnetic nanoparticles, the magnetism of the surface would not matter. Fun-tak was used to create a better seal between the pressure adapter and the flask. Even with 3D-printed rubber creating a friction fit seal around the neck of the flask, some air could still leak through. If the rubber seal was made to be too narrow, it risked breaking the flask. Thus, Fun-tak was useful in creating a seal and avoiding accidentally breaking the neck of the flask, which could create safety concerns in a laboratory setting. An example of this setup can be seen in Figure 11.

**Figure 12:**
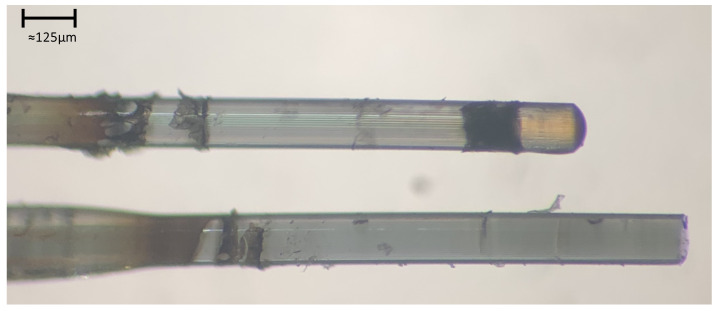
Confirmation of the full penetration of a colloidal nanocrystal solution completely drawn through the fiber using the new drawing process. The drawn PCF is on top (imaged from the end exposed to air during the drawing process), while an empty PCF is on the bottom for reference. Notice that the PCF with drawn solution has contrast to its air cores while the PCF without drawn solution is more opaque.

The magnetite was allowed to draw through the PCF for 23 h. Confirmation that the nanoparticle solution did indeed penetrate throughout the entire PCF was done by both visual inspection of the tip of the fiber (a small pool of magnetite could be seen on top of the fiber) and by using a microscope. This microscope image comparing a drawn PCF versus a non-drawn PCF can be seen in Figure 12.

A PCF fully drawn with nanomaterial can exhibit noticeable changes of its microchannels. This change is subtle, and the color of the colloidal solution may not be seen throughout the entire length of the fiber. The color of the solution can only be noticed at the end of the fiber due to the pooling of material. After drawing the magnetite nanoparticles through the PCF, the exterior of the PCF was cleaned using isopropyl alcohol and Kimwipes and then placed into a convection oven (model: Fisher Scientific Isotemp Oven, Fisher Scientific, Waltham, MA, USA). The solvent within the PCF was allowed to dry out over 72 h at 100 °C.

## 6. Three-Dimensional-Printed Fiber Connector Supporting Structure

**Figure 13:**
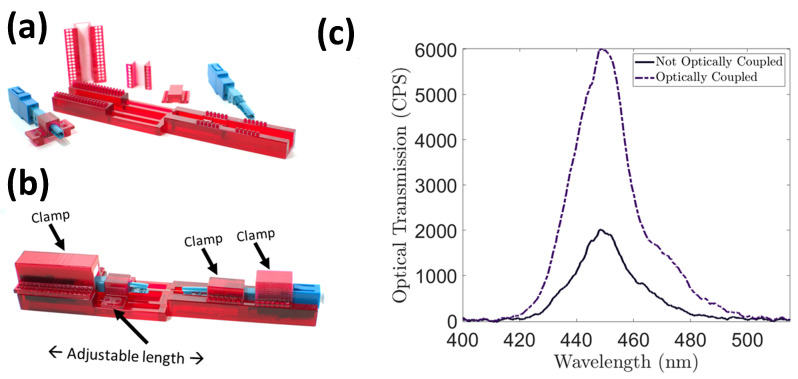
(**a**) The 3D-printed part with no clamps applied. (**b**) Fiber connectors with PCF in the structure with clamps applied. (**c**) Shows optical transmission before and after applying the correct pressure on the PCF fiber with the adjustable connector.

One of the unique aspects of this article was the development of a “clip-in” mechanism for the quick testing of PCF sensors. Usually in the literature, only a few PCF sensors can be tested due to the need to fusion splice the PCF sensor in order to be integrated into a test setup. While fusion splicing a fiber is the proper choice for a finalized sensor, it becomes burdensome when needing to compare different factors with repetitions. Hence, we created another 3D-printed part that securely held two LC fiber connectors and allowed for fine adjustment to one of the connectors in order to optimize the optical coupling of the PCF to the connectors. The 3D-printed parts consisted of a rubber material to allow for a secure clamp of the LC fiber connectors. The 3D-printed connector support structure can be seen in Figure 13. The orthographic drawings and cleaning process can be seen in the Appendix A.

The use of multi-material 3D printing allowed for easy scaling of the components. The fiber connectors used in our work were LC/PC connectors due to containing minimal metal components (connector spring). It also had a clip-on nature of an LC fiber-mating connector. However, SC/PC connectors can work as well. An area of improvement would be to modify LC/PC connectors with dielectric springs instead of metallic springs to expand its use for induction heating applications. To demonstrate the utility of this improved nanomaterial fiber integration methodology, an example experiment is shown in Appendix D.

## 7. Conclusions

We invented, created, and demonstrated a new way to cleave PCFs, draw nanomaterials into PCFs, and rapidly integrate PCFs sensors into a fiber-optic setup, thus achieving high reliability in cleaving PCF fibers without defects compared with cleaving with a diamond blade. From a previous study on conventionally cleaving PCFs, the improvement was quantified to be a 100% yield versus an ≈50% yield. This becomes important when drawing nanomaterials, as any capillaries with a defect on the PCF tip will prevent nanomaterials from being drawn. Next, the creation of a 3D-printed method to allow for drawing nanomaterials through multiple PCF fibers using positive nitrogen pressure decreased the amount of time one would need to draw over a dozen PCF fibers with varying types of nanofluid compositions. Previously, this drawing method would be done with one PCF at a time. Now, it can be done with at least three PCFs at a time, which reduces the processing time by threefold. In addition, the benefit of using positive pressure versus negative pressure is the assurance of safety, where one does not need to worry about liquid oxygen forming in their Schlenk line. Previously, others used an applied vacuum on the ends of the PCFs when drawing nanoparticles, which can create a potential safety risk if oxygen leaks into a Schlenk line [2,16]. Finally, a 3D-printed structure to hold the PCF sensors to allow for “clipping-in” to the optical system can allow for testing a large number of fibers with varying properties. This holds an advantage for measuring initial and approximate readings from new nanomaterial sensors when compared with the time-consuming process of fusion splicing. PCFs only need to be inserted into the clip-in apparatus, which takes approximately one minute to install and can be done in any laboratory versus needing to carefully align a PCF to MM fibers in a fusion splicer machine, which would take about an hour to go through the entire process. The structure allowed for adjustment to the amount of optical coupling through the LC connectors. A recommendation for future research would be to investigate the uniformity of the drawn nanoparticles within a PCF and how to best control the uniformity or determine the length limitation of a PCF integrated with a nanomaterial sensor.

## 8. Patents

USPTO Patent Pending #: 63/565,889—Laser Cleaving Plasma Cleaning Methodology for Reliable Cleaving of Glass Based Fibers.

USPTO Patent Pending #: 63/565,813—Magneto-Luminescent Nanomaterials Loaded Into Holey Fibers for Use In Radio Frequency Diagnostics.

## Figures and Tables

**Table 1:** Table of settings used to cut the PCF. The femtosecond laser was set to an output wavelength of 1035 nm and a pulse duration of 350 fs.

Test	RF Energy (μJ/pulse)	Repetition Rate (kHz)	Stage Speed (mm/s)
1	20	50	0.2
2	65	50	0.2
3	65	150	0.2
4	65	50	1
5	65	50	5
6	55	125	1

**Figure A1:**
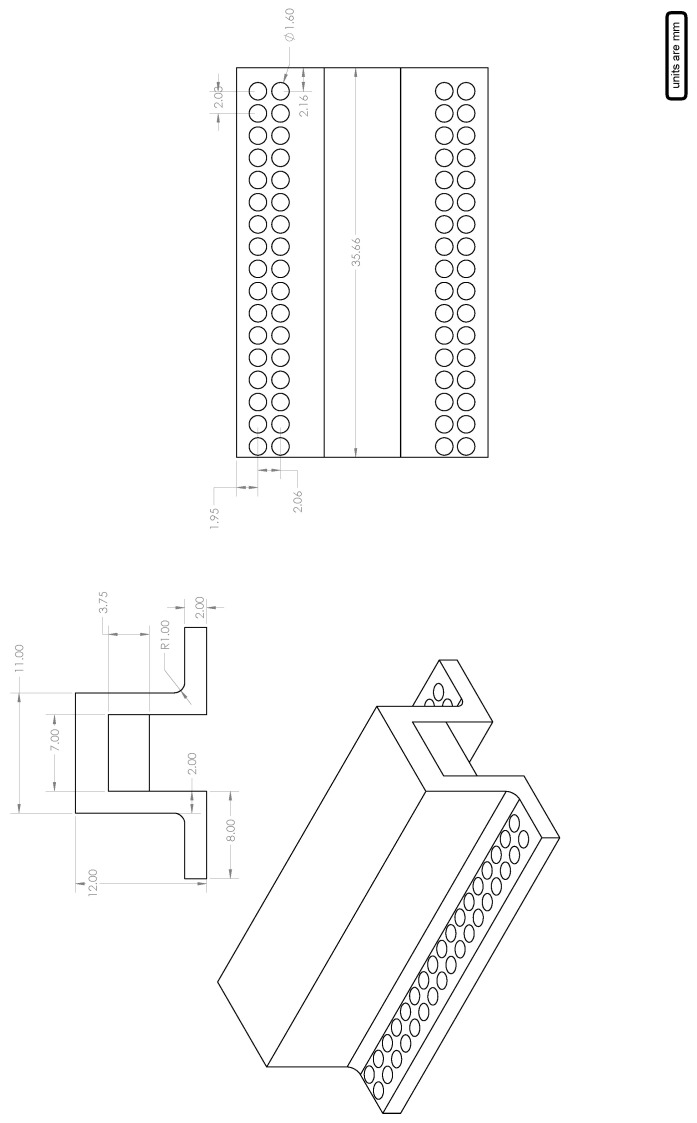
Orthographic drawing for the main clamp of the fiber connector structural support. The print time for one part is 50 min on high mix mode.

**Figure A2:**
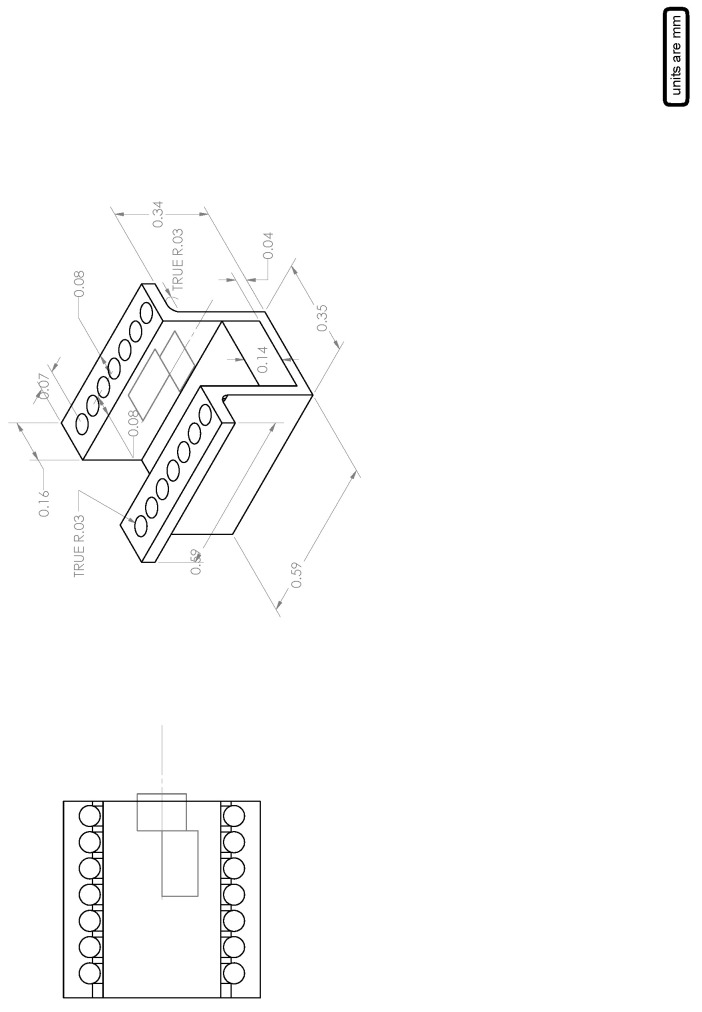
Orthographic drawing for the back clamp of the fiber connector structural support. The print time for one part is 42 min on high mix mode.

**Figure A3:**
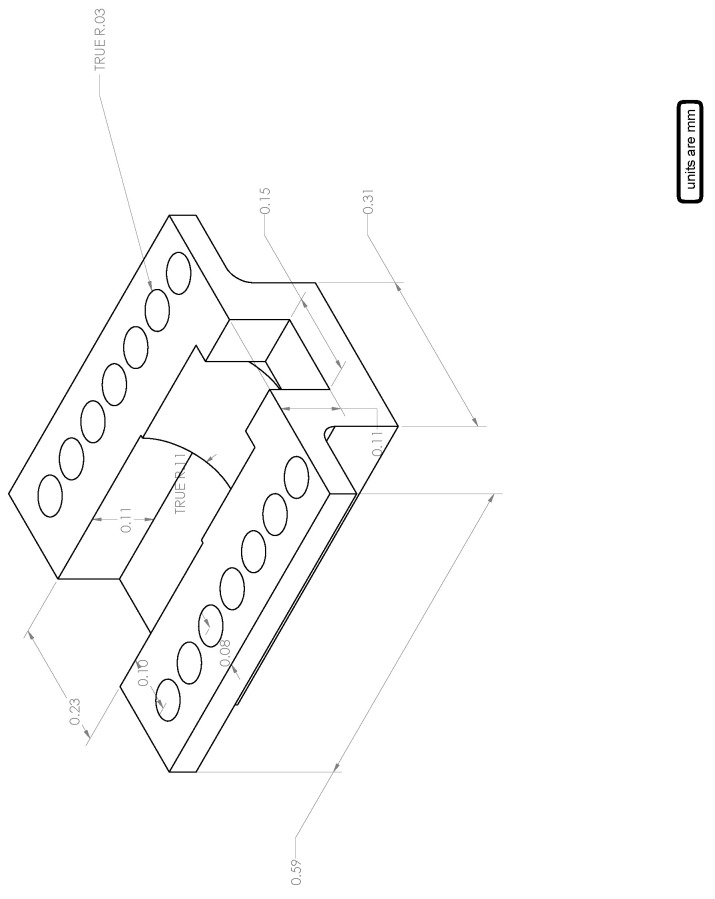
Orthographic drawing for the attachment clamp of the fiber connector structural support. The print time for one part is 22 min on high mix mode.

**Figure A4:**
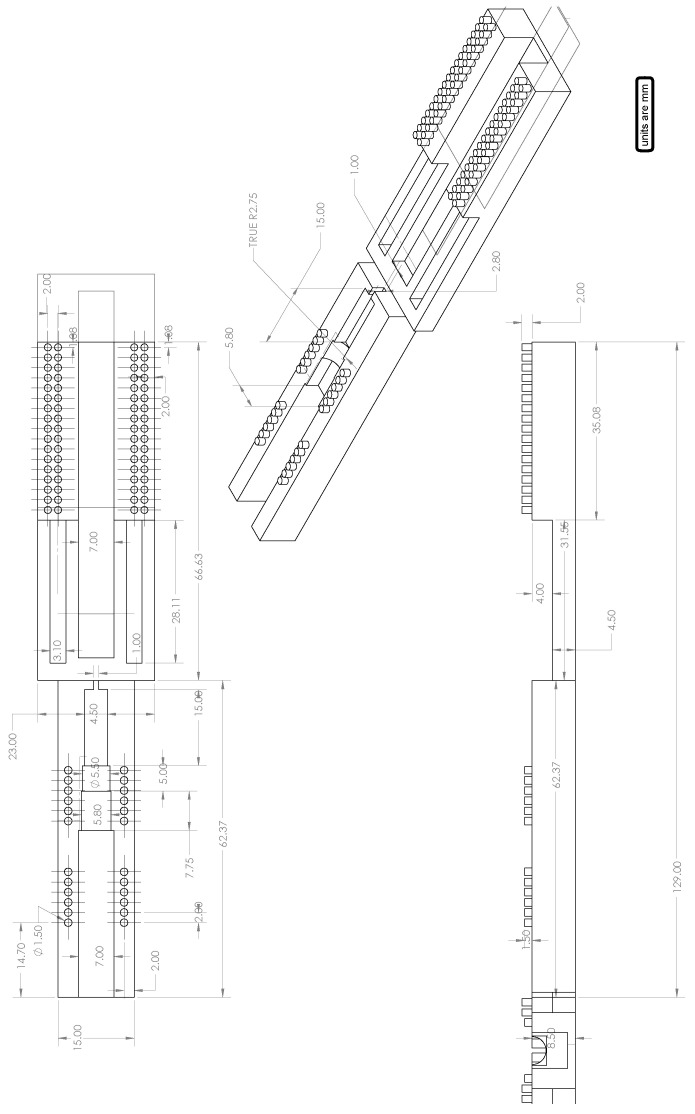
Orthographic drawing for the main body of the fiber connector structural support. The print time for one part is 52 min on high mix mode.

**Figure A5:**
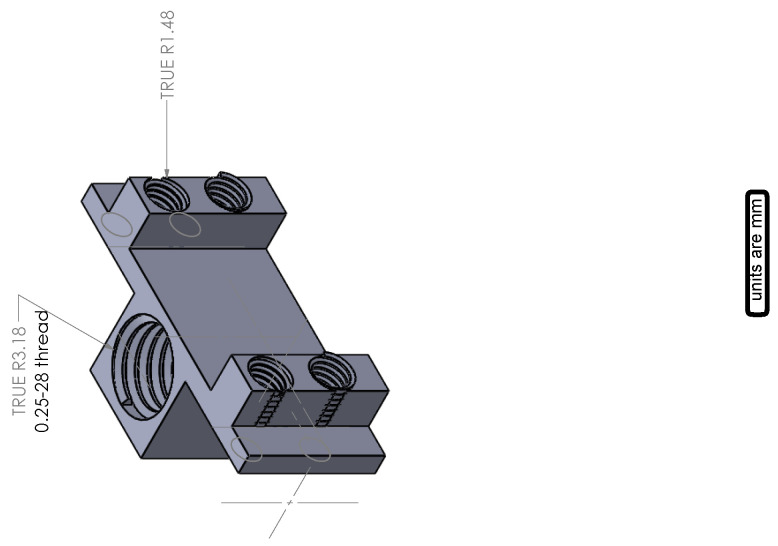

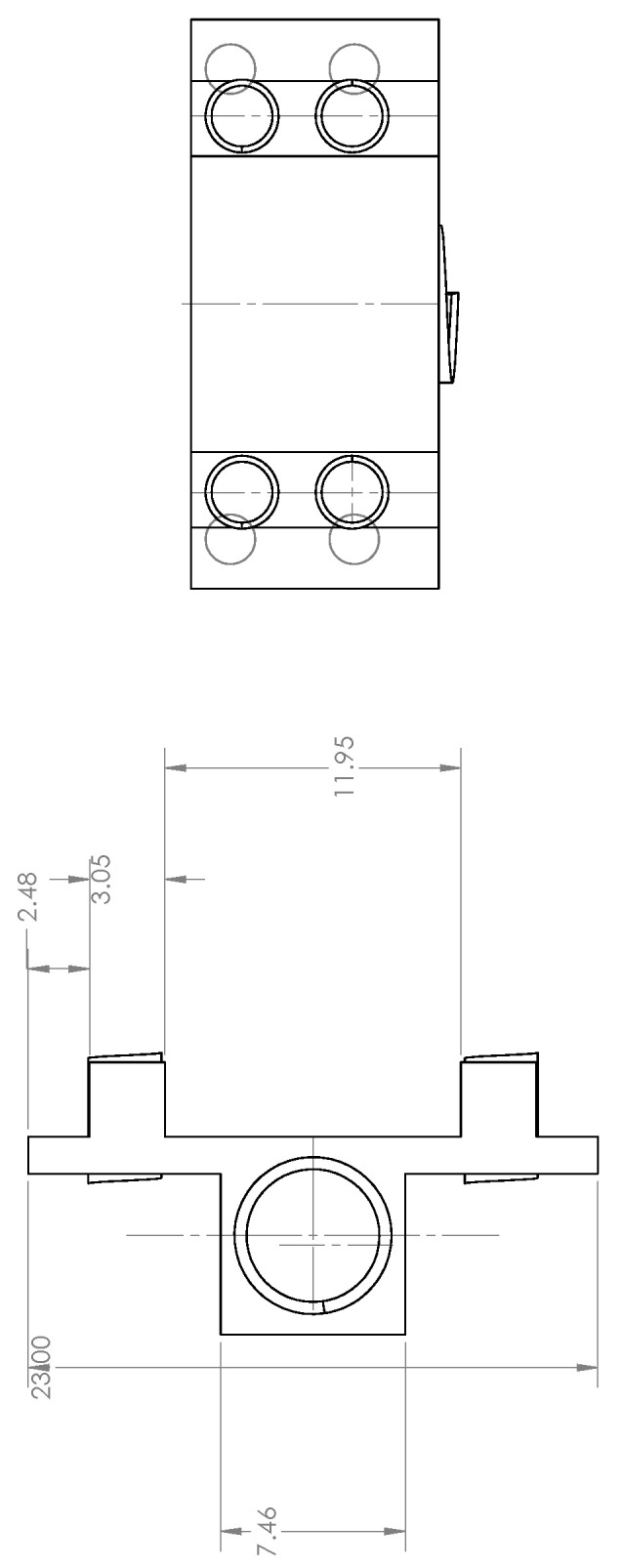
Orthographic drawing for the adjustable connector mount of the fiber connector structural support. The print time for one part is 43 min on high mix mode.

**Figure A6:**
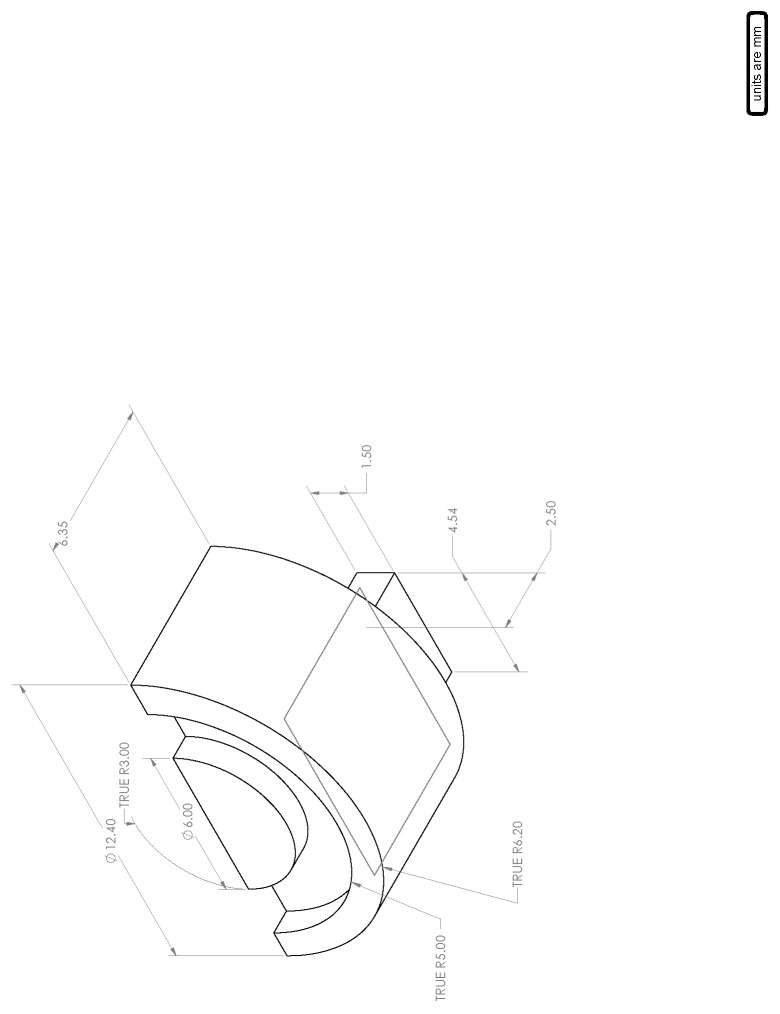
Orthographic drawing for the rubber clamps of the pressure adapter with low compression. One would use this for one side of the rubber clamp assembly. The print time for one part is 15 min on high mix mode.

**Figure A7:**
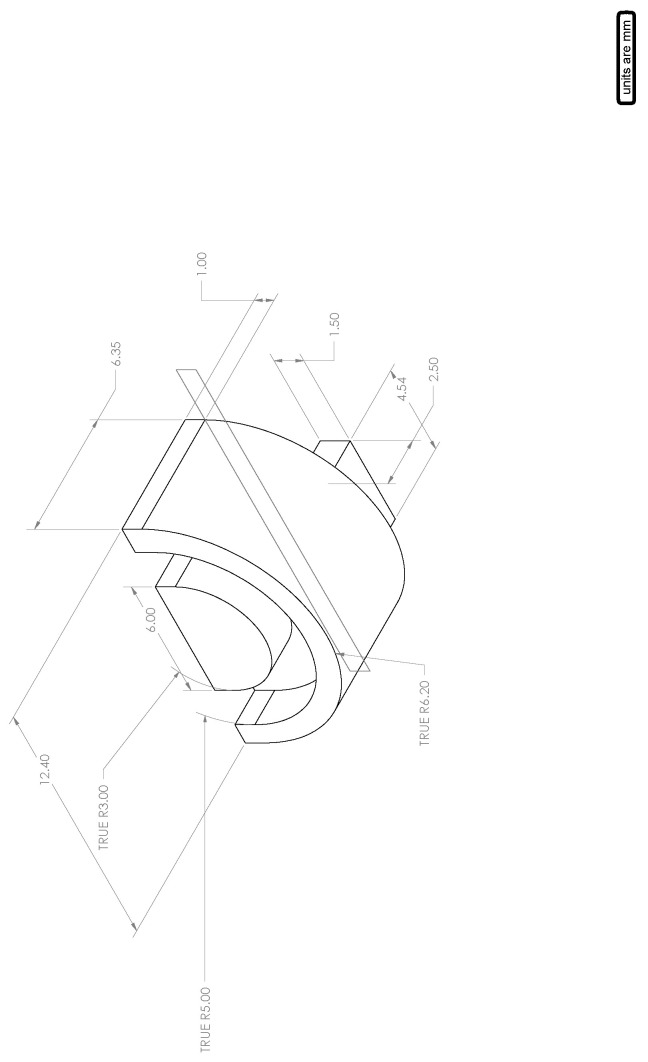
Orthographic drawing for the rubber clamps of the pressure adapter with high compression. One would use this for one side of the rubber clamp assembly. The print time for one part is 15 min on high mix mode.

**Figure A8:**
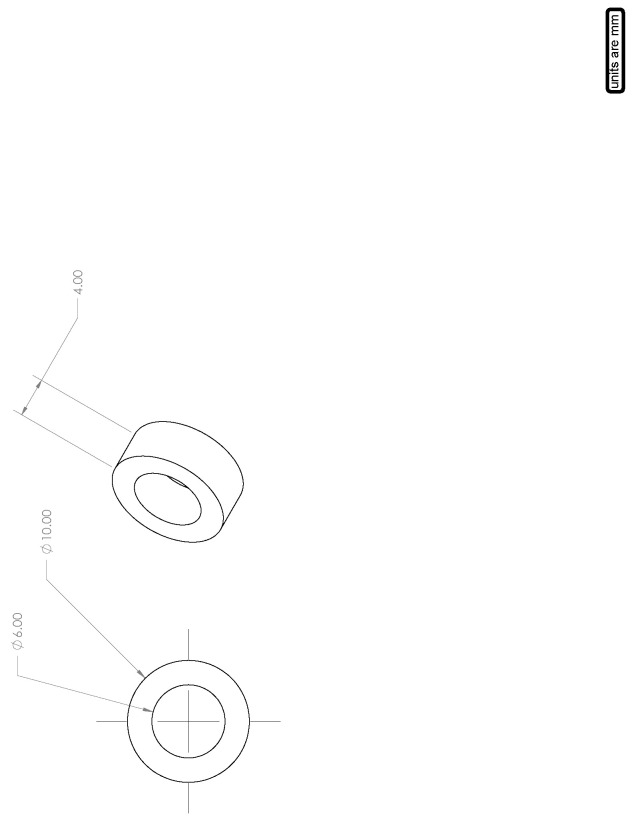
Orthographic drawing for the rubber o-ring of the fiber connector structural support. The print time for one part is 18 min on high mix mode.

**Figure A9:**
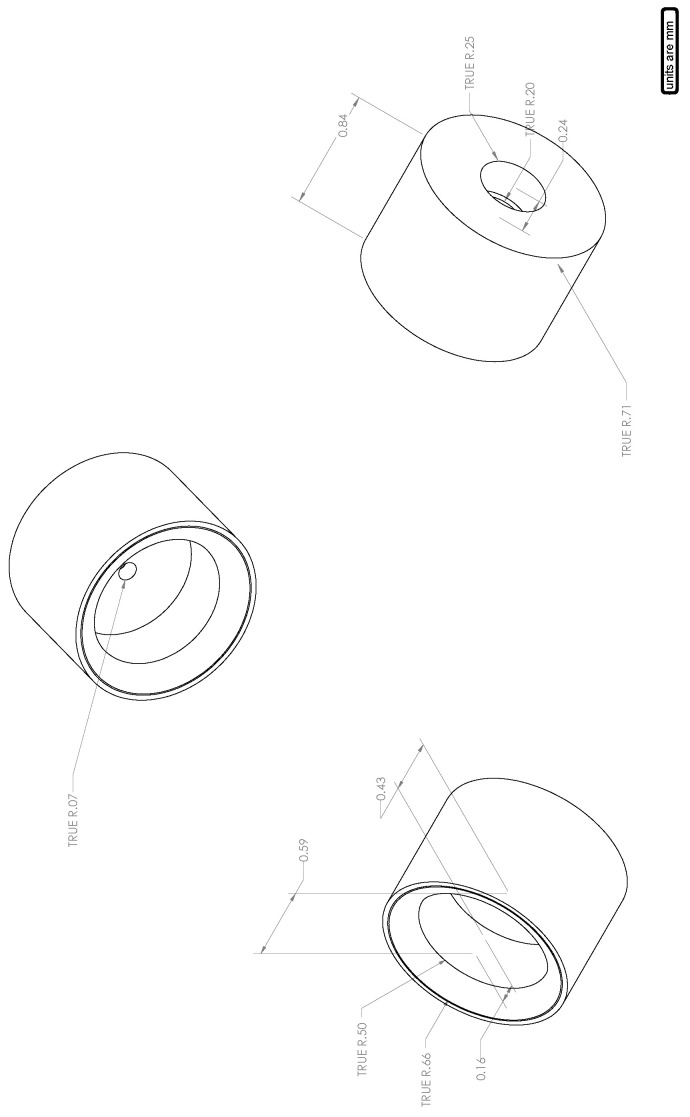
Orthographic drawing for the flask interface of the pressure adapter. The print time for one part is 1 h 53 min on high mix mode.

**Figure A10:**
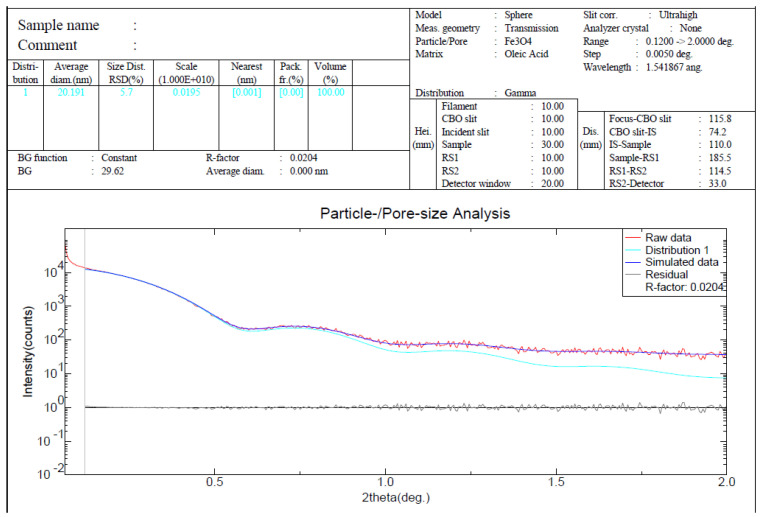
SAXS curve of 20.2 nm diameter NPs.

**Figure A11:**
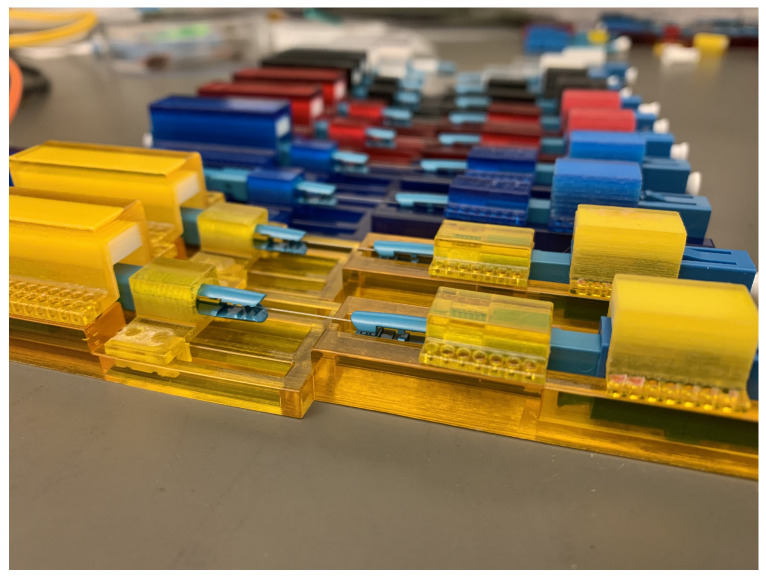
Visual showing the scaled production of PCFs drawn with magnetite nanocrystals and inserted into the clip-in fiber mechanism for an initial experiment.

**Figure A12:**
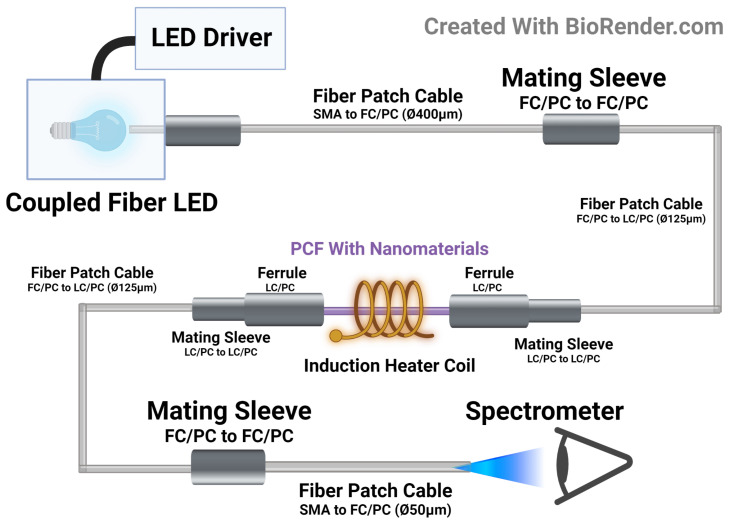
Illustration of the experimental setup. The optical source was an LED that was then coupled to a fiber. This then transmitted through the entire fiber system, which contained the magnetite-nanocrystal-loaded PCF. The optical transmission through the PCF was sensed with an optical spectrometer. An induction heater was placed around the PCF to supply both an electromagnetic field and a heating source.

**Figure A13:**
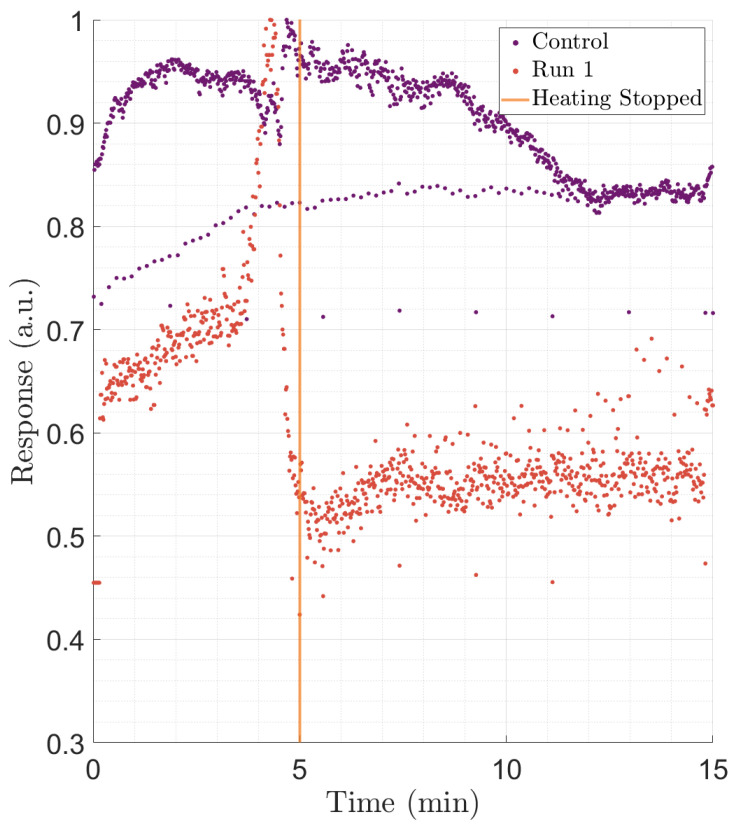
Changes in optical spectra of run and control at 40 mT.

**Figure A14:**
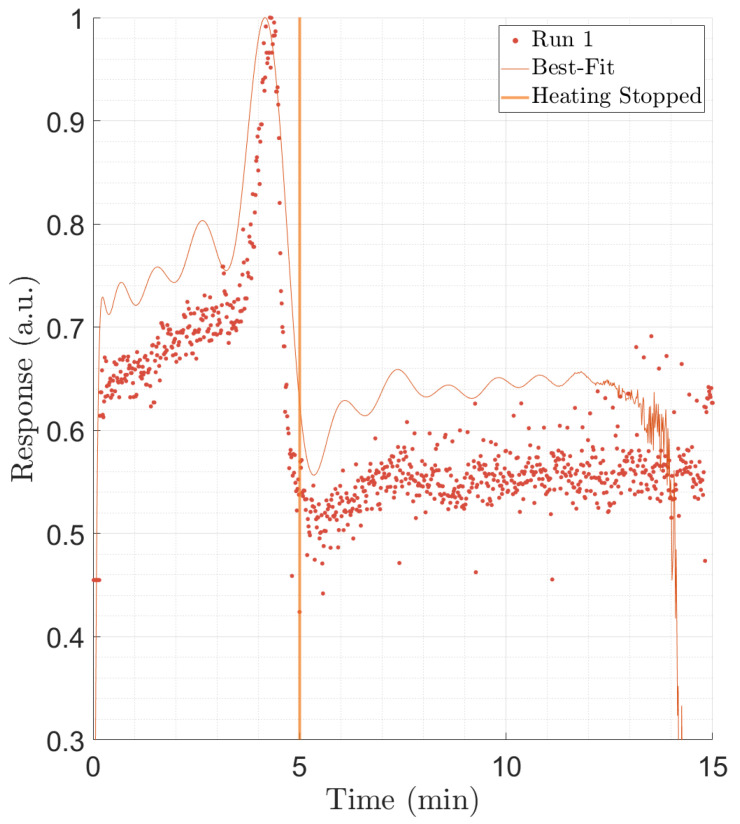
Best-fit line of scatter plot.

**Figure A15:**
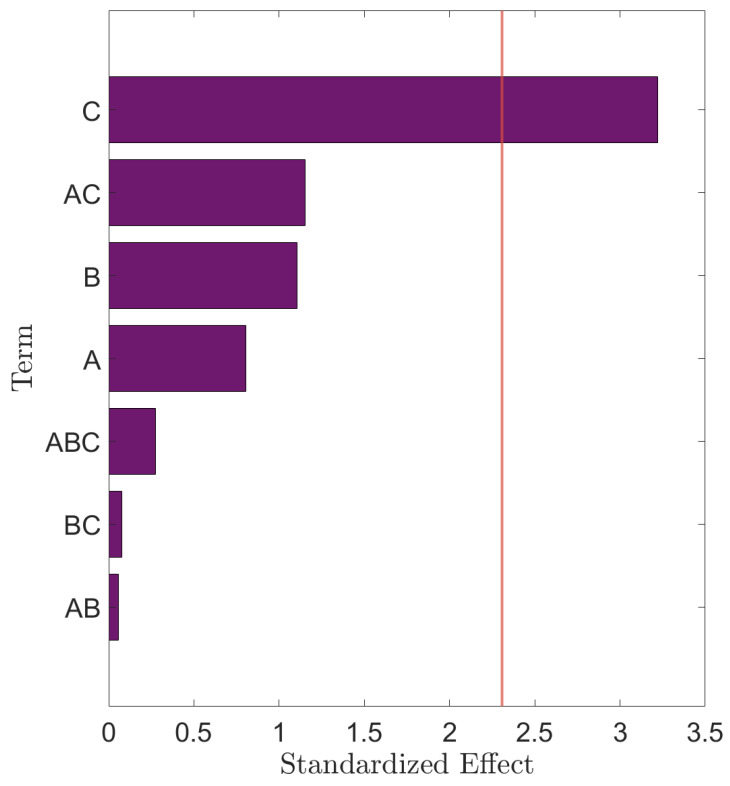
Pareto chart showing significant factors.

## Data Availability

Data are contained within the article.

## Abbreviations

The following abbreviations are used in this manuscript:

ANOVA	Analysis of variance
CQD	Colloidal quantum dots
DoEx	Design of experiments
LC	Lucent connector
LCPC	Laser-cleaving plasma cleaning
LED	Light-emitting diode
MM	Multimode
MO	Magneto-optic
NIR	Near infrared
SA	Saturable absorber
SAXS	Small-angle X-ray scattering
SEM	Scanning electron microscope
SMA	SubMiniature Version A
SNR	Signal-to-noise ratio
PCF	Photonic crystal fiber
PFIB	Plasma-focused ion beam
PVA	Polyvinyl alcohol
TEM	Transmission electron microscope
TGA	Thermogravimetric analyzer
TIR	Total internal reflection
USB	Universal serial bus
XRD	X-ray diffraction

## Appendix D. Initial Induction Heater Magnetite Nanocrystal Experiment Using New Photonic Crystal Fiber Processing Methodology

The primary intent of this paper was to demonstrate improved ways to process PCFs for integration with nanocrystals intended for sensor use cases. A major advantage of the methodology proposed in this paper is the ability to scale up the processing. This is visually highlighted in Figure A11. The clip-in mechanism made it relatively easy to fusion splice the PCFs to MM fibers to integrate 10 PCFs for experimentation. This section goes over an experiment that looked into magnetite nanocrystals of two different diameters and two different concentrations for possible uses as magneto-optic (MO) sensors when used in an induction heating application.

### Appendix D.1. Experimental Setup

As illustrated in Figure A12, a light-emitting diode (LED) driver (model: Thorlabs LEDD1B, Thorlabs, Newton, NJ, USA) using a power supply (model: Thorlabs KPS101, Thorlabs, Newton, NJ, USA) was used to power a fiber-mated 455 nm LED (model: Thorlabs M455F3). This fiber-mated LED was connected to a 1 m long SubMiniature Version A (SMA) to FC/PC fiber patch cable (model: Thorlabs M76L01). This was then connected to a fiber-mating sleeve (model: Thorlabs ADASMA), which was connected to a 9/125 μm FC/LC fiber patch cable. This was connected to an LC-to-LC mating connection with the end connected to an LC ferrule. The PCF was connected to the LC ferrule on both ends. The other end of the PCF was connected to another LC-to-LC mating connection to an FC/LC fiber patch cable. This connected to a fiber-mating sleeve (model: Thorlabs ADASMA) and connected to an FC/SMA fiber patch cable (model: Thorlabs M76L01). Finally, this patch cable connected to a universal serial bus (USB) spectrometer (model: Ocean Optic FLAME-S-VIS-NIR-ES, Ocean Insight, Orlando, FL, USA).

Design of experiments (DoEx) was used to test three factors: (a) the average diameter of the magnetite nanoparticles, (b) the concentration of the magnetite nanoparticles in the colloidal solution, and (c) the magnetic field applied. Two levels were applied to each factor. For factor A, the diameters were 7 nm and 19.4 nm. For factor B, the concentrations were 5 $\frac{\mathrm{mg}}{\mathrm{mL}}$ and 50 $\frac{\mathrm{mg}}{\mathrm{mL}}$. For factor C, the magnetic fields were 30 mT and 40 mT. It was determined from preliminary testing that these sensors needed ≈30 mT to show any meaningful optical changes, thus the low level was set to this value. Three replications were done in order to bound the variance due to the fabrication process and the integration into the fiber testing system. This led to a total of 16 runs.

The design table used for conducting the experiment in this study can be seen in Table A1. The fiber number column shows the fabricated fiber repetition used for that run. For example, there were two manufactured high-diameter and high-concentration magnetite PCFs. These were then alternated throughout the runs whenever a high-diameter and high-concentration run came up in order to consider the variance in the manufacturing process.

The output used for studying the changes between the factors and levels was the change in the optical transmission intensity through the fiber. The magnetic field was applied for 5 min. After turning off the magnetic field, the optical transmission was measured for another 10 min. The optical transmission data were collected every 5 s with a 1 s integration time.

The two-level, three-factor experiment provided data to establish conclusions on whether the concentration of iron oxides or the diameter of iron oxides had a larger effect on the optical transmission response, whether the effect of the concentration and the diameter of the iron oxides were coupled to the size of the magnetic field, to understand the role magnetic heating played in the change in the optical transmission response, and to obtain a generalized trend for further optimization of this MO sensor.

**Table A1:** Experimental design with two repetitions showing the run order for three factors and their two respective levels. The positive (+) and negative (-) levels in the table corresponds to the higher and lower values for each factor respectively.

Run	A	B	C
1	-	+	+
2	+	-	+
3	-	-	+
4	-	-	+
5	+	+	+
6	-	+	-
7	-	-	-
8	+	+	-
9	-	+	-
10	+	+	-
11	+	-	-
12	+	-	-
13	+	+	+
14	+	-	+
15	-	-	-
16	-	+	+

### Appendix D.2. Discussion and Results

The optical transmission data showed a unique trend over both the heating and cooling processes. An optical transmission scatter plot from one of the runs at 40 mT and the control at 40 mT can be seen in Figure A13.

Upon turning on the induction heater, the optical peak transmission intensity immediately dropped for the first minute before rising for the remainder of the heating process. Upon turning off the induction heater, the optical transmission intensity continued to rise for another two minutes, most likely from residual heat. At this point, the system began to cool and caused a discontinuity jump that drastically lowered the optical transmission intensity, which then continued to descend over time. After a certain point, the optical transmission intensity slowly recovered back to its original state (in ≈10 min). This trend could be seen across all runs. The discontinuity effect could be seen in the control case, and thus, this was attributed to the heating of the metal crimp attached to the LC fibers. In order to assess the runs with less bias, best-fit line polynomial functions of a high-order could be applied after removing the extraneous/outlier data points. Due to the noise of the scatter plots (some of the PCFs had a lower amount of light transmitted through them, which was most likely due to the amount of nanocrystals drawn/non-uniform draw); these best-fit lines were only approximate due to varying SNRs of the data collected for each run. An example can be seen in Figure A14.

Next, all of the data points taken for each run from the best-fit lines between 1.67 min and 13.33 min had their medians taken. Taking the medians within the middle data points mitigated the data error from the best-fit lines toward the edges of the plot. These medians were then compared in the DoEx analysis.

### Appendix D.3. Experimental Measurement Results

The ANOVA table for this experiment can be seen in Table A2.

Table A2 shows that the magnetic field strength had the most contribution to the overall signal change. The two-way interaction between both the diameter of the magnetite nanocrystals and the magnetic field strength was the next-highest contributor toward the overall signal change. However, this contribution percentage for the two-way interaction and everything else with a lower contribution percentage was not conclusive. This was because the overall error of the experiment was 36.93%. This high experimental error could be attributed to the following: varying transmission optical signal strengths noticed for each of the different combinations of magnetite diameters and concentrations, the best-fit line technique used to establish a baseline through signals with a low SNR, and the heating of the metal ferrule of the LC fiber going into the 3D-printed PCF MO sensor. The results in Table A2 are summarized in a Pareto chart in Figure A15, which shows which factor was statistically significant at a 95% confidence interval.

**Table A2:** ANOVA table showing the contributions of each factor for the experiment. Factor A is the diameter of the nanoparticles, factor B is the concentration, and factor C is the magnetic field strength. DF is the number of degrees of freedom used for each model.

Source	DF	Contribution (%)
**Model**	7	63.07
**Linear**	3	56.56
A	1	2.99
B	1	5.63
C	1	47.94
**2-Way Interactions**	3	6.16
AB	1	0.01
AC	1	6.12
BC	1	0.03
**3-Way Interactions**	1	0.34
ABC	1	0.34
**Error**	8	36.93
**Total**	15	100.00

Since the magnetic field strength was the only factor with significance, the main phenomenon that influenced the results was from the metal crimps of the LC fibers. Despite these results being deemed inconclusive for a magnetite-nanocrystal-based MO sensor, the strength of the proposed processing methodology proposed in this paper can be seen. A large number of PCF sensors were made and could be used to effectively use DoEX in order to statistically determine whether a desired experimental study carries significance. If there is no significance, one can more effectively use the significance of each factor to determine what in the experiment went wrong. In this case, it was discovered that the metal spring in the LC connector was not advertised as being part of the product.
